# Changes in pain following bilateral intermittent theta-burst, transcranial magnetic stimulation for depression: A retrospective chart review

**DOI:** 10.1080/24740527.2023.2300026

**Published:** 2024-01-12

**Authors:** Sawmmiya Kirupaharan, Roumen Milev, Joanne Bressee, Sonya Kelso, Scott Duggan, Felicia Iftene, Tim V. Salomons, Wilma Hopman, Ian Gilron

**Affiliations:** a Faculty of Health Sciences, Queen’s University, Kingston, Ontario, Canada; b Department of Psychiatry, Queen’s University, Kingston, Ontario, Canada; c Department of Psychology, Queen’s University, Kingston, Ontario, Canada; d Providence Care Hospital, Kingston, Ontario, Canada; e Department of Anesthesiology and Perioperative Medicine, Queen’s University, Kingston, Ontario, Canada; f Department of Public Health Sciences, Queen’s University, Kingston, Ontario, Canada

**Keywords:** chronic pain, depression, transcranial magnetic stimulation, neuromodulation

## Abstract

**Introduction:**

Pain management in patients with chronic pain and comorbid depression is challenging and understudied. There is interest in intermittent theta-burst stimulation (iTBS), a new modality of repetitive transcranial magnetic stimulation (rTMS). This retrospective review describes changes in pain, anxiety and depression throughout iTBS treatment at the dorsolateral prefrontal cortex (DLPFC).

**Methods:**

A retrospective chart review was conducted of patients who underwent their first acute series of iTBS treatments at the DLPFC for depression at a single institution between 2020 and 2023. Data on depression, anxiety, and pain were collected throughout iTBS treatment using the Beck Depression Inventory–II (BDI-II; higher scores indicate worse depression) and visual analogue scale (VAS; 0–100, higher scores indicate worse pain, anxiety, and depression). Nonparametric tests were used for all analyses.

**Results:**

Of 104 patients, 52 reported moderate pain at baseline (50.0%). Median BDI-II scores decreased from 38.0 (interquartile range [IQR] = 29.0–44.0) to 24.0 (IQR = 9.0–36.0) from pre- to posttreatment (*P* < 0.001). Of the 32 patients with both pre- and posttreatment pain scores, there was a significant decrease from 40.0 (IQR = 5.5–71.8) to 15.0 (IQR = 3.5–53.8; *P* = 0.037). In patients with at least moderate pain at baseline, pain scores decreased from 71.0 (IQR = 55.0–80.0) to 20.0 (IQR = 11.0–71.0; *P* = 0.004). Ten of 32 patients with available pre- and posttreatment scores reported ≥30% reduction in pain scores (31.2%).

**Conclusion:**

These preliminary results, suggesting decreases in pain following iTBS treatment, provide a rationale for future rigorous investigations to evaluate this intervention for depression and comorbid chronic pain.

## RÉSUMÉ

**Introduction:** La prise en charge de la douleur chez les patients souffrant de douleur chronique et de dépression comorbide est difficile et sous-étudiée. Il existe un intérêt pour la stimulation thêta-burst intermittente (STBi), une nouvelle modalité de stimulation magnétique transcrânienne répétitive (SMTr). Cette revue rétrospective décrit les changements dans la douleur, l’anxiété et la dépression tout au long du traitement par STBi au niveau de la région du cortex préfrontal dorsolatéral.Un examen rétrospectif des dossiers a été mené pour les patients ayant reçu leur première série intensive de traitements par STBi pour la dépression dans la région du cortex préfrontal dorsolatéral dans un seul établissement entre 2020 et 2023. Les données sur la dépression, l’anxiété et la douleur ont été collectées tout au long des traitements par STBi à l’aide de l’Inventaire de dépression de Beck-II (IDB-II; des scores plus élevés indiquent une dépression plus grave) et de l’échelle visuelle analogique (EVA; 0-100, des scores plus élevés indiquent une aggravation de la douleur, de l’anxiété et de la dépression). Des tests non paramétriques ont été utilisés pour toutes les analyses.

**Résultats:** Parmi les 104 patients inclus dans l’étude, 52 ont déclaré une douleur modérée au départ (50,0 %). Les scores médians de l’IDB-II ont diminué de 38,0 (intervalle interquartile [IQR] = 29,0-44,0) à 24,0 (IQR = 9,0-36,0) avant et après le traitement (P < 0,001). Parmi les 32 patients pour lesquels des scores de douleur avant et après le traitement étaient disponibles, une diminution significative a été constatée, passant de 40,0 (IQR = 5,5-71,8) à 15,0 (IQR = 3,5-53,8 ; P = 0,037). Chez les patients hospitalisés avec une douleur au moins modérée au départ, les scores de douleur ont diminué de 71,0 (IQR = 55,0-80,0) à 20,0 (IQR = 11,0-71,0 ; P = 0,004). Dix des 32 patients pour lesquels des scores avant et après le traitement étaient disponibles ont rapporté une réduction ≥30 % des scores de douleur (31,2 %).

**Conclusion:** Ces résultats préliminaires, qui indiquent une diminution de la douleur après le traitement par STBi, offrent une justification pour la réalisation de futures études rigoureuses afin d’évaluer cette intervention pour la dépression et les douleurs chroniques comorbides.

## Background

Chronic pain and depression frequently coexist, with longitudinal studies suggesting that nondepressed individuals with pain have a significantly higher risk of developing depression over time.^1–3^ In comparison to patients with isolated pain or depression, patients with comorbid pain and depression report worse outcomes, such as amplified and persistent pain and greater functional limitation.^4,5^ However, this complex population is often undertreated and often excluded from clinical trials of treatment interventions.^6,7^ Patients with depression are less likely to receive medical attention for their coexisting pain than nondepressed patients.^8^ Further, the management of patients with chronic pain and comorbid depression is challenging. Response to standard analgesic or antidepressive drug therapy is often poorer in patients with preexisting depression and pain, respectively.^9,10^ As such, there is growing interest in identifying effective and safe treatments for the management of comorbid chronic pain and depression.

Repetitive transcranial magnetic stimulation (rTMS) is a noninvasive neuromodulation technique that involves the delivery of a precise, localized magnetic field to parts of the brain through a coil placed on the scalp.^11,12^ Stimulation parameters for rTMS treatments, including the frequency, intensity, and duration of the pulses—as well as anatomical site of stimulation—vary depending on treatment indication. rTMS pulses modulate neural activity in the brain and produce a site-specific functional change. Although treatment mechanisms continue to be investigated, rTMS targeting the dorsolateral prefrontal cortex (DLPFC) of the brain has proven to be a safe and effective treatment for the management of medication-resistant depression.^13^ Current evidence suggests that stimulation of the DLPFC may provide both antidepressant and analgesic effects, making it a promising target for the treatment of patients with depression and comorbid chronic pain.^14^ However, clinical trials assessing the analgesic and antidepressant effect of rTMS often exclude patients with primary psychiatric diagnoses, limiting our ability to make conclusions about the effectiveness of this treatment modality in this more complex population.^15^

Novel rTMS stimulation protocols are gaining interest. Intermittent theta-burst stimulation (iTBS) is a newer rTMS modality involving the delivery of pulses higher in frequency to standard rTMS protocols, mimicking theta rhythms in the brain.^16^ Early evidence suggests that iTBS protocols have comparable efficacy for depression and superior efficacy for pain in comparison to standard rTMS protocols, while also improving cost-effectiveness and reducing treatment time.^17,18^ However, there is a paucity of evidence on the effectiveness of this new protocol on concurrent pain and depression.

As such, there is impetus to study the effectiveness of rTMS, specifically iTBS protocols, in the concurrent treatment of pain and comorbid depression. This retrospective chart review therefore aims to describe changes in pain, anxiety, and mood following a patient’s first acute series of iTBS treatments and explore the association between changes in mood and pain.

## Methods

### Patients

A retrospective review of medical health records of patients who underwent their first acute series (25–30 consecutive treatments) of iTBS treatments for the indication of depression at a single institution (Providence Care Hospital, Kingston, Ontario, Canada) between January 2020 and January 2023 was conducted. Patients included in this review received rTMS treatment as bilateral iTBS of the DLPFC using the Cool B70 coil Mag Pro magnetic stimulator according to the parameters outlined in the following section. All patients who were treated during the study time period were included in this review and no patients were excluded from treatment based on presence or severity of pain or the severity of depression at baseline. Ethics approval for this retrospective review was obtained from the Queen’s Health Sciences and Affiliated Hospitals Research Ethics Board (ANAE-389-22). Informed consent was not sought from patients due to the retrospective and low-risk nature of this study.

### iTBS Treatment Protocol

Consent for iTBS treatment and TMS Adult Safety Screen was completed by a psychiatrist prior to treatment.^19^ Contraindications to rTMS treatment included history of cerebrovascular conditions, seizures, or implanted metallic hardware. Medications known to impact the neuronal excitability (e.g., pregabalin, gabapentin) were contraindicated during iTBS treatment.

Resting motor threshold (RMT) was obtained at first treatment and as needed thereafter by applying single stimuli over the primary motor cortex (M1) using the minimum intensity required to produce a muscle twitch in the contralateral hand. RMT measurement was repeated if the patient had medication changes during treatment, changes in response, or treatment-emergent side effects. The DLPFC, which corresponds to the F3 location, was then located using the International 10–20 system and the location was marked on a cap.^20^ The cap was used to aid in the consistent identification of the F3 location on subsequent treatments. The intensity of rTMS pulses was started at 80% of RMT and was titrated up to 120% of RMT as tolerated. An acute series of rTMS treatments is ordered as 25 or 30 treatments in total and could be adjusted as required depending on the treatment tolerability and response. The 25-treatment series was delivered once daily on weekdays for 5 weeks; patients receiving 30 treatments received 2 treatments daily for a total of 3 weeks.

iTBS treatment was delivered over the right and left DLPFC using the following parameters: Duration of 3:09 min on each side; amplitude: 1.0; mode: standard; waveform: biphasic burst; burst pulses: three; interpulse interval: 20 ms; repetition rate: 5 pulses per second; train pulse: 10; delay: 8 s; trains: 20.

### Data Collection

Baseline demographic data, including patient age and sex, and concurrent antidepressant, anti-anxiety, and analgesic medications that were prescribed to patients during their iTBS treatment were ascertained through chart review. The primary outcomes of this review included self-reported ratings of mood, anxiety, and pain from baseline to the end of iTBS treatment. The Beck Depression Inventory–II (BDI-II)^21^ was completed by patients before treatment and immediately following the last treatment of their first acute series. The BDI-II consists of 21 items that are each scored from 0 to 3, with a higher number indicating a greater experience of depressive symptoms. Visual analogue scale (VAS; 0–100) ratings of mood, anxiety, and pain were rated at pretreatment; at treatment sessions 6, 11, 16, and 21; and immediately following the last treatment in the series. Mid-treatment scores were collected after the treatment was delivered. Patients were asked to rate their pain, mood, and anxiety on the VAS scale from 0 to 100, with high scores indicating worse mood, anxiety, and pain. Patients with pain at baseline were then asked for reasons regarding their pain, including chronic and acute pain. Patients with a VAS pain score from 10 to 30 were considered to have mild pain, from 40 to 60 moderate pain, and from 70 to 100 severe pain.^22^ Due to the retrospective nature of this study, data collection was not standardized and therefore there was a risk of missing data. Missing data in this study does not reflect attrition of patients in the iTBS treatment.

### Data Analysis

Data analysis was completed using IBM SPSS v28.^23^ A majority of outcome data were nonnormally distributed according to the Shapiro-Wilk test (Supplementary Table A). As such, all outcome data are reported as medians with interquartile ranges (IQRs). Categorical variables are reported as frequencies and percentages. Exploratory comparisons between pre- and posttreatment depressive symptoms, mood, anxiety, and pain scores were conducted using the paired Wilcoxon signed rank test and reported as *Z* statistics with *P* values and the effect size, *r*. Using the benchmarks for Cohen’s effect sizes, an *r* value of 0.2 was considered a small effect and 0.5 and 0.8 were considered moderate and large effect sizes respectively. Subgroup analyses were conducted in patients who reported at least moderate pain at baseline (VAS ≥40). This analysis excludes patients with no pain or mild pain at baseline, who were less likely to demonstrate meaningful analgesic effect.^22^

In the subset of patients with a complete set of data, the Friedman test, a nonparametric repeated measures analysis of variance test, was conducted and corresponding *P* values are reported. Given significant missing data, a series of Mann-Whitney *U* tests was conducted to compare pretreatment pain, depression, mood, and anxiety scores in patients (1) with and without posttreatment scores and (2) with and without a complete data set. This was done to assess for any substantial baseline differences between participants who did and did not complete posttreatment assessment and between those who did and did not provide data at all time points. Exploratory correlational analyses between changes in pain and changes in depressive symptoms were completed using the Spearman’s correlation for nonparametric data and are reported as Spearman’s correlation coefficient, rho. A *P* value <0.05 was considered statistically significant and no adjustment was made for multiple comparisons.

## Results

### Patient Characteristics

A total of 104 patients were included in this retrospective review (70.2% females), with an average age of 48.4 (SD = 13.7). Of these patients, 73 received 25 treatments in their first acute series of iTBS treatments (70.2%) and 31 received 30 treatments (29.8%). Table 1 displays concurrent antidepressant, anti-anxiety, and analgesic medications that were prescribed to patients during their iTBS treatment. Of the 58 patients with concurrent medication data available, 33 were prescribed antidepressants, 40 were prescribed anti-anxiety medications, and 16 were prescribed analgesics. At baseline, 52 patients reported at least moderate pain (VAS ≥40; 50.0%). Twenty-two of the patients in this cohort reported having a chronic pain condition (21.2%): 10 did not specify their chronic pain diagnosis (9.6%), 6 reported fibromyalgia (5.8%), 4 reported chronic back pain (3.8%), 1 reported chronic migraine (0.9%), and 1 reported chronic temporomandibular joint pain (0.9%).

**Table 1.** Demographic characteristics of included patients.

Age	48.4 (13.7)
Sex (reference: female)	73 (70.2%)
Antidepressants (*n* = 56)	
Bupropion	11
Trazadone	8
Brexpiprazole	7
Quetiapine	7
Mirtazapine	5
Duloxetine	4
Venlafaxine	4
Sertraline	3
Vortioxetine	2
Lithium carbonate	2
Imipramine	1
Desipramine	1
Fluoxetine	1
Moclobemide	1
Nortriptyline	1
Phenelzine	1
Lurasidone	1
Anti-anxiety (*n* = 56)	
Clonazepam	20
Lorazepam	16
Citalopram	2
Diazepam	2
Escitalopram	1
Analgesics (*n* = 56)	
Hydromorphone	4
Oxycocet	3
Acetaminophen/codeine	3
Naproxen	2
Morphine	2
Oxycodone	1
Fentanyl patch	1
Codeine	1
Tramadol	1

### Changes in Mood and Anxiety

BDI-II scores and mood and anxiety VAS scores throughout iTBS treatment are displayed in Supplementary Figures A to C. Patients reported a median pretreatment BDI-II score of 38.0 (IQR = 29.0–44.0; *n* = 91) and a posttreatment BDI-II score of 24.0 (IAR = 9.0–36.0; *n* = 61; Table 2). Of the 54 patients who had both pre- and posttreatment BDI-II scores available, there was a statistically significant decrease in scores from 37.5 (IQR = 28.0–43.3) to 24.5 (IQR = 9.8–36.0; *Z* = −5.074, *P* < 0.001, *r* = −0.49; Table 3).

**Table 2.** Outcomes reported throughout iTBS.

	*n*	BDI-II scores, median (IQR)	*n*	VAS mood, median (IQR)	*n*	VAS anxiety, median (IQR)	*n*	VAS pain, median (IQR)
Pretreatment	91	38.0 (29.0–44.0)	100	72.0 (50.0–80.0)	100	65.0 (31.0–80.0)	100	41.0 (5.5–72.0)
Session 6			70	65.0 (43.8–80.0)	70	54.5 (30.0–75.0)	70	45.0 (15.0–68.5)
Session 11			72	62.0 (39.3–77.5)	72	55.0 (30.0–75.0)	72	40.0 (15.0–61.5)
Session 16			57	50.0 (30.0–70.5)	57	50.0 (20.0–70.0)	57	30.0 (00.0–64.5)
Session 21			45	49.0 (25.0–66.5)	45	42.0 (16.5–65.0)	45	35.0 (10.0–55.5)
Posttreatment	61	24.0 (9.0–36.0)	34	35.0 (11.5–66.3)	34	24.5 (11.5–66.3)	34	16.5 (4.5–51.3)

**Table 3.** Pre- and posttreatment comparisons of depressive symptoms, mood, anxiety, and pain.

	*n*	Pretreatment scores, median (IQR)	Posttreatment scores, median (IQR)	Wilcoxon signed rank test	Effect size, *r*
BDI-II	54	37.5 (28.0–43.2)	24.5 (9.8–36.0)	*Z* = −5.074, *P* < 0.001	−0.49
VAS mood	32	75.0 (56.3–80.0)	35.0 (12.0–68.8)	*Z* = −4.433, *P* < 0.001	−0.55
VAS anxiety	32	65.0 (31.3–81.5)	21.5 (10.5–68.8)	*Z* = −4.002, *P* < 0.001	−0.50
VAS pain	32	40.0 (5.5–71.8)	15.0 (3.5–53.8)	*Z* = −2.085, *P* = 0.037	−0.26
VAS pain scores in patients with moderate pain at baseline	17	65.0 (50.0–84.0)	20.0 (11.0–71.0)	*Z* = −2.844, *P* = 0.004	−0.49

Patients reported a median mood VAS score of 72.0 (IQR = 50.0–80.0; *n* = 100) to 35.0 (IQR = 11.5–66.3; *n* = 34) from pre- to posttreatment. Mood scores (*Z* = −4.433, *P* < 0.001, *r* = −0.55) and anxiety scores (*Z* = −4.002, *P* < 0.001, *r* = −0.50) decreased significantly from pre- to posttreatment in the 32 patients who had both pre- and posttreatment data available.

Sixteen patients had a complete set of data for mood and anxiety scores (i.e., pretreatment; sessions 6, 11, 16, 21; and posttreatment scores). Friedman’s repeated measures analysis of variance revealed statistically significant differences across timepoints in mood (*P* < 0.001) and anxiety (*P* = 0.006).

### Changes in Pain

**Figure 1.**
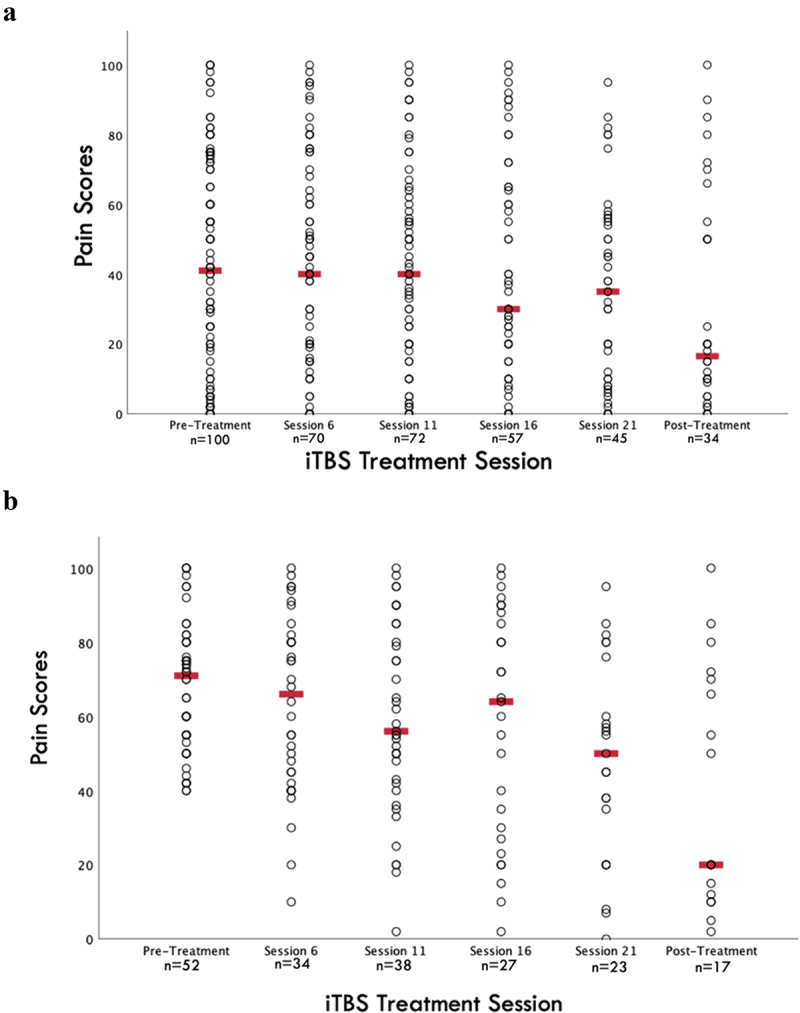
Scatterplot of visual analogue scale pain scores over intermittent theta-burst stimulation treatment for a) the full cohort, and b) the subgroup of the cohort with at least moderate pain at baseline. Circles represent raw patient pain scores; red lines represent median pain scores at each time point.

Pain scores reported throughout iTBS treatment are displayed in Figure 1a. At pretreatment, patients reported a median pain score of 41.0 (IQR = 5.5–72.0; *n* = 100) and 16.5 (IQR = 4.5–51.3; *n* = 34) at posttreatment.

Thirty-two patients of the full cohort of 104 patients had both pre- and posttreatment data available (30.8%). Of the 32 patients, there was a significant decrease from 40.0 (IQR = 5.5–71.8) to 15.00 (IQR = 3.5–53.8; *Z* = −2.085, *P* = 0.037, *r* = −0.26), and 10 patients experienced a ≥30% decrease (31.2%). Of the 16 patients with all data points available, the Friedman repeated measures analysis of variance demonstrated a *P* = 0.072.

Subgroup analyses of patients who reported at least moderate pain at baseline (VAS ≥40) demonstrated significantly lower pain scores from pre- to posttreatment (Figure 1b; *Z* = −2.844, *P* = 0.004, *n* = 17, *r* = −0.49). Patients reported an average pretreatment score of 71.0 (IQR = 55.0–80.0; *n* = 52) and a posttreatment score of 20.0 (IQR = 11.0–71.0; *n* = 17). Pre- and posttreatment mood (*Z* = −3.148, *n* = 17, *P* = 0.002, *r* = −0.54), anxiety (*Z* = −2.794, *n* = 17, *P* = 0.005, *r* = −0.48), and BDI scores (*Z* = −3.690, *n* = 29, *P* < 0.001, *r* = −0.48) also decreased significantly in this subgroup of patients.

### Attrition Bias

Given significant missing data at the posttreatment time point, analyses were conducted to compare pretreatment pain, mood, anxiety and BDI scores in patients with and without posttreatment data, demonstrating nonstatistically significant differences (*P* = 0.909, *P* = 0.320, *P* = 0.547, and *P* = 0.167, respectively, on Mann-Whitney *U* test). Similarly, comparative analyses were conducted in pretreatment pain, mood, and anxiety scores in the 16 patients with a complete data set for all time points and 84 patients with missing data. This series of analyses demonstrated nonstatistically significant results with *P* = 0.788, *P* = 0.314, and *P* = 0.818 for pain, mood, and anxiety, respectively, on Mann-Whitney *U* test. BDI data were only collected pre and post, so this additional analysis was not relevant for this outcome.

### Correlational Analyses

**Figure 2.**
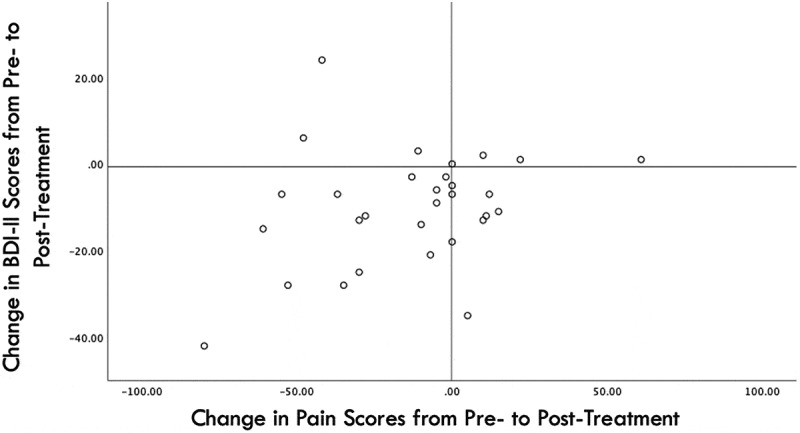
Scatterplot of changes in pain and changes in BDI scores from pre- to posttreatment.

Of the 30 patients with available data for all outcomes, 15 reported concurrent decreases in pain, mood, anxiety, and BDI scores. Spearman’s correlation between changes in pain and changes in BDI-II scores yielded a positive, nonsignificant correlation (rho = 0.246, *P* = 0.189; Figure 2).

## Discussion

There is an evolving body of evidence on the safety and efficacy of rTMS in the treatment of chronic pain conditions including fibromyalgia, migraines, and peripheral neuropathic pain.^24^

The majority of clinical trials of rTMS on comorbid pain and depressive symptoms have notably excluded patients with primary psychiatric diagnoses. In a meta-analysis conducted on rTMS in patients with fibromyalgia pain, despite reporting depressive symptoms as a secondary outcome in most of the included studies, all trials excluded patients with a major depressive disorder.^15^ One sham-controlled study that evaluated the analgesic effect of stimulation of the DLPFC in patients with major depressive disorder suggested analgesic and antidepressive effects.^25^ However, details regarding participants’ pain history and diagnosis were not reported. As such, despite promising results, the ability to make firm conclusions on the concurrent effect of rTMS for depression on pain based on these studies is limited.

Our study found statistically significant decreases in pain scores following iTBS of the DLPFC. Subgroup analyses of patients who reported at least moderate pain at baseline demonstrated a more substantial decrease in pain scores, with a nearly moderate effect size of 0.49. Interestingly, this effect size was comparable to that of the BDI-II scores from pre- to post-iTBS treatment. Of note, 31.2% of patients with available data experienced a reduction in pain scores ≥30%, which was considered a clinically significant difference.^26^

The site of rTMS delivery is a key moderator of its effectiveness in providing analgesic benefit. Current evidence-based guidelines on the use of rTMS recommend the stimulation of M1 for analgesia in patients with neuropathic pain and the stimulation of the DLPFC for antidepressant treatment.^27^ Though some literature suggests that there is efficacy in rTMS of the DLPFC in reducing pain, recent evidence suggests greater analgesic efficacy when targeting the motor cortex (M1).^28,29^ In addition, though some data suggest that, of the regions of the brain accessible by rTMS therapy, the motor cortex is the most susceptible to inducing seizures, recent clinical evidence suggests similar safety profiles in the stimulation of the DLPFC and M1, with the incidence of adverse events reported by patients being comparable in both groups.^29,30^

However, although there is greater analgesic effect when targeting the M1, the concurrent antidepressive effect of this treatment is minimal.^25,31^ There were no significant improvements in BDI scores from pre- to posttreatment in randomized controlled trials evaluating the effect of rTMS of the M1 in fibromyalgia and neuropathic pain.^25,32,33^ On the other hand, there is growing interest in the study of rTMS of the DLPFC on both depression and pain symptoms.^14^ In subgroup analyses of a meta-analysis, Hou et al.^14^ suggested that stimulation of the M1 was effective at reducing pain and fatigue and improving general health and function. Similar to our study, Hou et al.^14^ also found that stimulation of the DLPFC was effective at reducing pain and depression and improving general health and function, suggesting its utility in the treatment of chronic pain and depression.

The mechanisms through which rTMS of the DLPFC impacts pain remain unclear. Current theories include the generation of a direct neuromodulatory effect on pain (e.g., modulation of activity of descending pain circuits) and pain-related cognitive pathways, indirect analgesic effects secondary to antidepressive effects, or a combination of both pathways.^24,34–36^ Though our study demonstrated a significant decrease in both pain and BDI scores, a positive, nonsignificant correlation between changes in pain and changes in depression was demonstrated. Although it is not possible to make conclusions based on this level of evidence, this finding is interesting in the context of the potential mechanisms through which iTBS of the DLPFC may impact pain and depression. Multicenter randomized controlled trials are required to validate the findings of our study and further explore the mechanisms through which rTMS of the DLPFC reduces pain, in conjunction with basic science research.

Further, this study investigated the effectiveness of a newer rTMS protocol that used higher frequency pulses over a shorter treatment session, known as the iTBS protocol. The iTBS protocol has been shown to mimic theta rhythms in the brain, contributing to its comparable, if not greater, effects compared to standard rTMS protocols.^37^ In addition to added clinical benefit, the shorter duration of iTBS sessions contributes to significant cost savings per patient.^38^ Though studies have investigated the effectiveness of iTBS in isolated depression and chronic pain, to our knowledge, trials have not yet studied the potential for this treatment modality to treat concurrent pain and depression.

Consistent with estimates of the prevalence of pain in patients with depression, our study found that 50% of our retrospective cohort with major depressive disorder had moderate concurrent pain. Surveys in outpatient settings of patients with depression suggest a prevalence of pain ranging from 59% to 65%.^5,39^ Given the significant comorbidity of these conditions, the development of adequate screening protocols in primary care settings is vital to identifying and adequately treating patients in this population.^40,41^ Further, though 21.2% of patients volunteered a chronic pain diagnosis, this may be an underestimate of the prevalence of chronic pain in our cohort.

The results of this study, however, were affected by a substantial amount of missing data. At this institution, data on pain, mood, and anxiety were initially gathered for the purpose of monitoring treatment-emergent side effects and therefore the collection of these data was not part of a standardized protocol. Comparative analyses of pretreatment scores in patients with and without posttreatment scores and patients with and without complete data sets were conducted to evaluate the potential for attrition bias. This series of analyses did not demonstrate any statistically significant differences between groups with missing data and those without. Although these findings lessen the possibility of missing data owing to attrition bias, we are unable to conclusively determine the reasons for significant missing outcome scores.

Further, though syntheses of randomized controlled trials have consistently demonstrated a significant clinical effect of rTMS on neuropathic pain, a recent meta-analysis of randomized controlled trials revealed that rTMS in patients with fibromyalgia did not produce a significant analgesic effect.^42–44^ Though beyond the scope of this study, future studies should evaluate the type of chronic pain syndrome as a moderator of analgesic effect. Also, maintenance treatments of rTMS may be key in producing optimal analgesic and antidepressive effects. Mhalla et al., for example, demonstrated that monthly rTMS treatments following the first acute series of treatments contributed to an analgesic effect that was maintained for 6 months.^31^ As such, future studies should report the number of maintenance treatments a patient required and conduct long-term follow-up assessments.

### Limitations

This study is not without limitations. Firstly, the lack of a control group in this study limits our ability to assess the true effect of this intervention on pain. Further, although this study demonstrated statistically significant improvements in pain, mood, and anxiety, these analyses were limited by the small sample with complete pre and post data. Due to the retrospective nature of the study, it was not possible to determine the reasons for the significant missing data. Further, some patients received antidepressant, anti-anxiety, and analgesic medications during their iTBS treatment period that we were unable to account for in our descriptive analyses. This is largely because we did not have an accurate record of the total doses of medications taken by patients over their iTBS series or a list of over-the-counter analgesics. Chronic pain diagnoses were volunteered by patients in this study and therefore might not be reflective of the true prevalence of chronic pain in this population. Also, reasons for changes in pain throughout iTBS treatment (e.g., new acute pain, treatment-emergent pain, chronic pain) were not consistently ascertained. Due to the limited sample size of patients with chronic pain in this study, we were unable to perform subgroup analyses and explore the analgesic effect of rTMS on patients with different types of chronic pain syndromes and different lengths of rTMS treatments (i.e., 25 or 30 treatments). Because this was a descriptive, retrospective data review and not a prospective observational study (or randomized controlled trial), no primary endpoint could be designated for this study. Additionally, other secondary outcomes that are vital to understanding the effectiveness of chronic pain interventions including sleep quality and quality of life were not ascertained. These limitations should be addressed in future through robust prospective studies.

## Conclusion

The results of this study contribute to the evolving body of literature on rTMS, specifically iTBS protocols, for the concurrent treatment of depression and comorbid chronic pain. Patients undergoing their first acute series (25–30 consecutive treatments) of iTBS reported lower pain scores following treatment. Though these findings suggest that iTBS for depression may improve pain, rigorously conducted prospective double-blind controlled trials are required to confirm these findings and to understand potential treatment mechanisms in this complex group of patients.

## Supplementary Material

Supplemental Material

Supplemental Material

Supplemental Material

Supplemental Material
