# Supplementary figures and images for: Changes in pain following bilateral intermittent theta-burst, transcranial magnetic stimulation for depression: A retrospective chart review
Source: Can J Pain. 2024 Jan 12;8(1):2300026. doi: 10.1080/24740527.2023.2300026 (PMC10936632; doi:10.1080/24740527.2023.2300026)

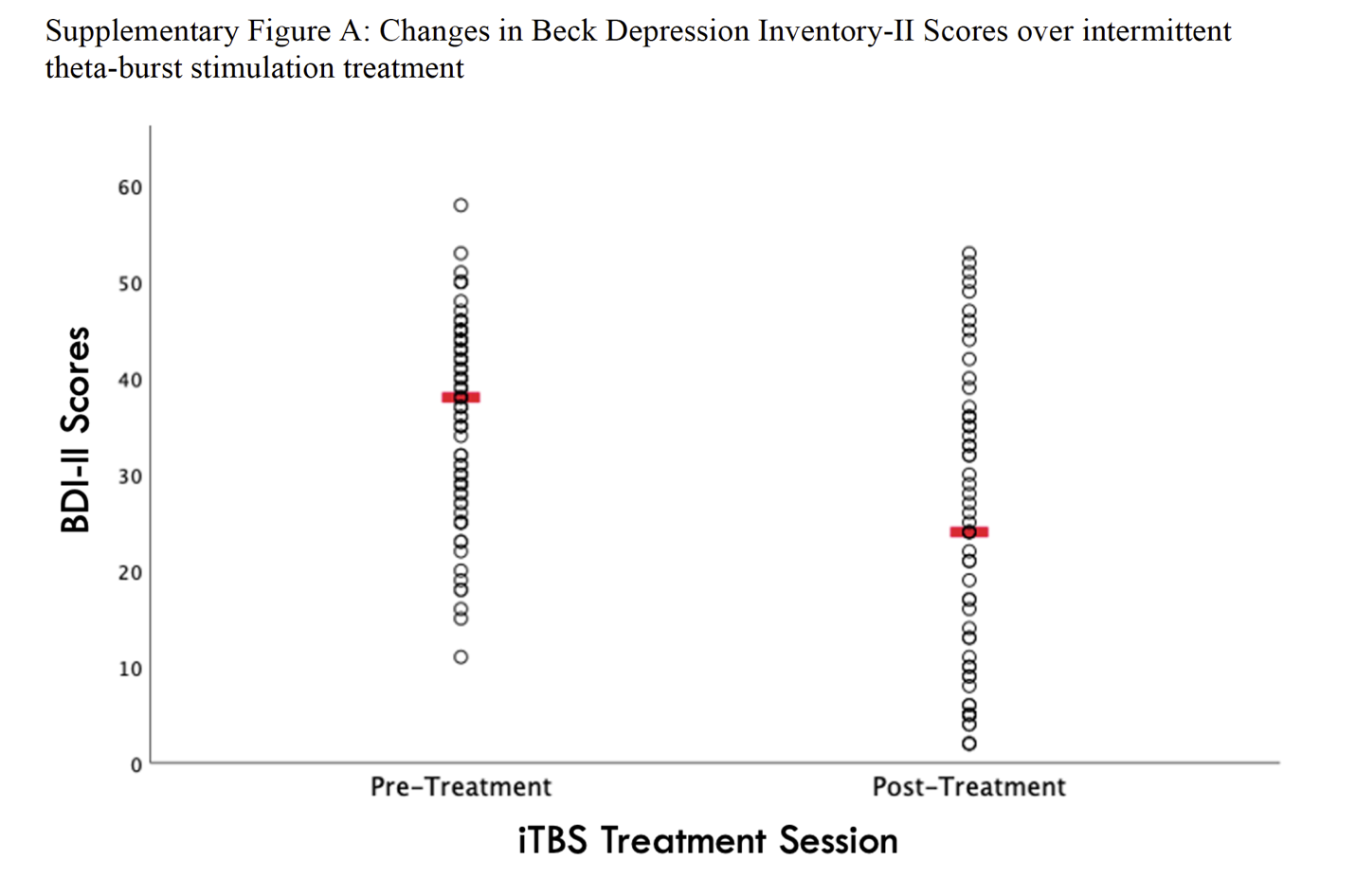

Supplement: Supplemental Material [file UCJP_A_2300026_SM1782.docx]

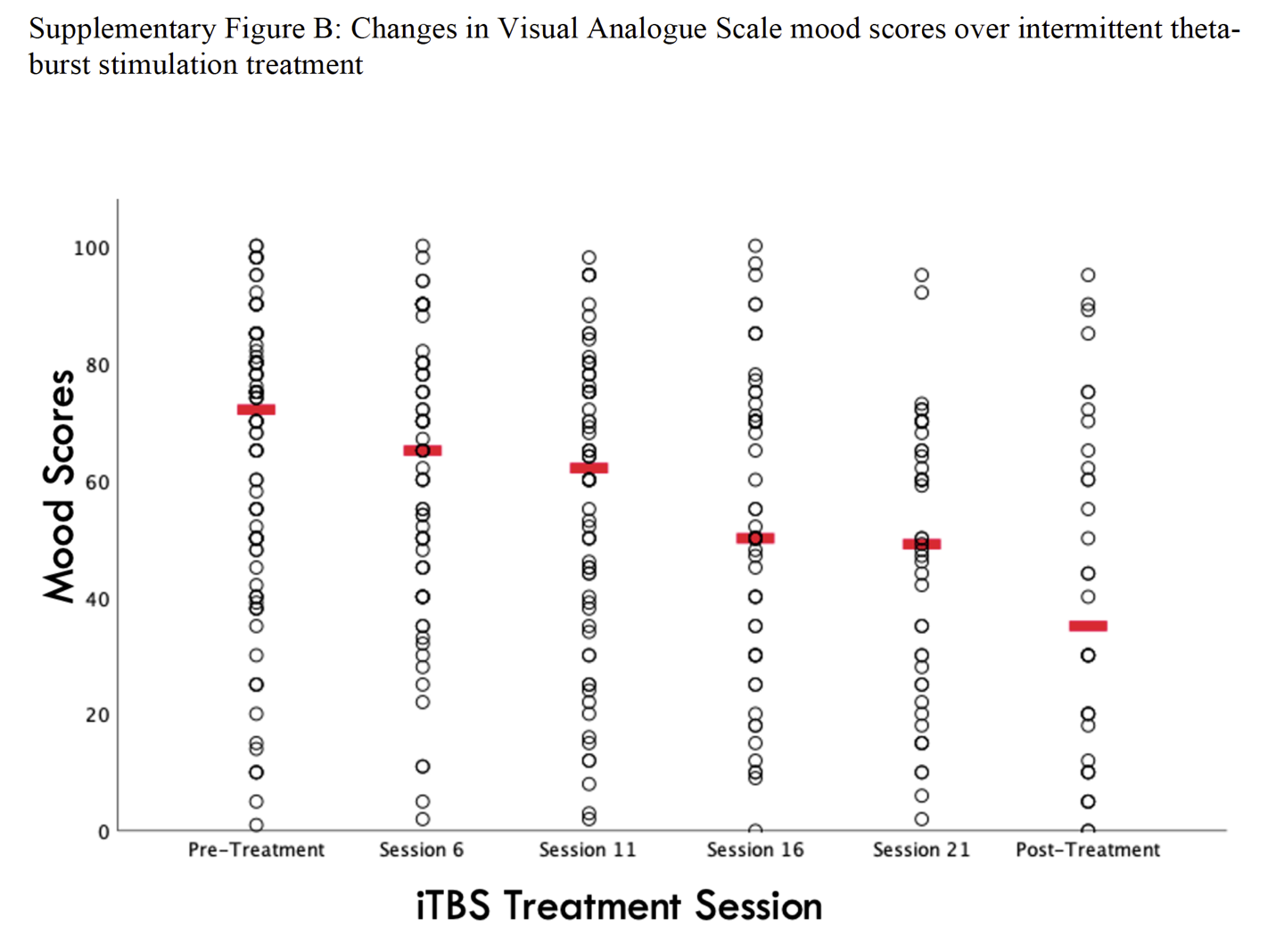

Supplement: Supplemental Material [file UCJP_A_2300026_SM1776.docx]

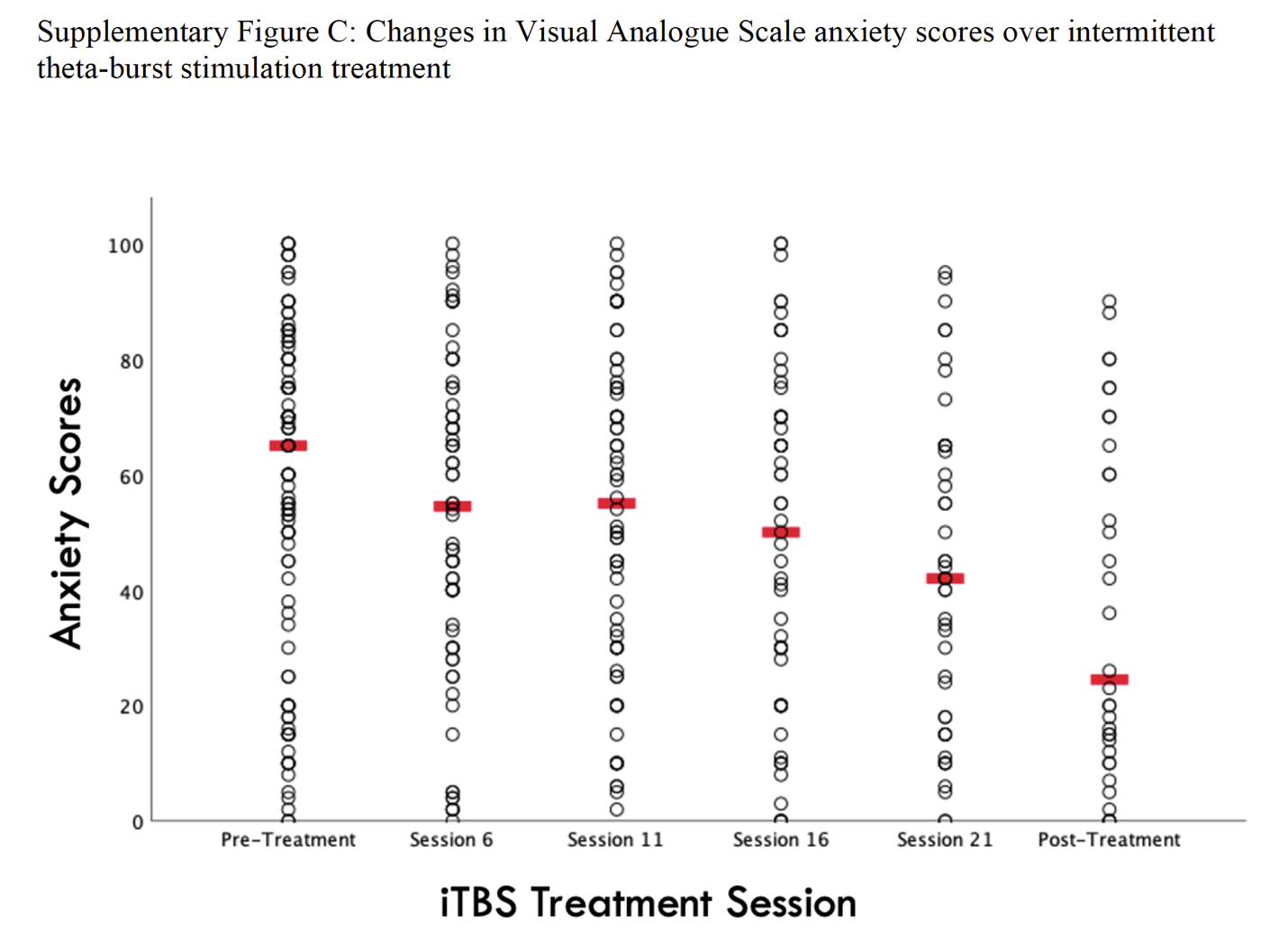

Supplement: Supplemental Material [file UCJP_A_2300026_SM1774.docx]
