# Supplementary material for: Changes in pain following bilateral intermittent theta-burst, transcranial magnetic stimulation for depression: A retrospective chart review
Source: Can J Pain. 2024 Jan 12;8(1):2300026. doi: 10.1080/24740527.2023.2300026 (PMC10936632; doi:10.1080/24740527.2023.2300026)
Supplement: Supplemental Material [file UCJP_A_2300026_SM1772.docx]

Supplementary Table A: Shapiro-Wilk test for normality.

|  | Statistic | Degrees of Freedom | p-value |
| --- | --- | --- | --- |
| Pre-treatment BDI | 0.97 | 91 | 0.057 |
| Post-treatment BDI | 0.94 | 61 | 0.004 |
| Pre-treatment Pain | 0.91 | 100 | <.001 |
| Post-treatment Pain | 0.83 | 34 | <.001 |
| Pre-treatment Mood | 0.93 | 100 | <.001 |
| Post-treatment Mood | 0.93 | 34 | 0.032 |
| Pre-treatment Anxiety | 0.93 | 100 | <.001 |
| Post-treatment Anxiety | 0.90 | 34 | 0.004 |
